# Fatigue and Fracture Behavior of a Cold-Drawn Commercially Pure Aluminum Wire

**DOI:** 10.3390/ma9090764

**Published:** 2016-09-08

**Authors:** Jia-Peng Hou, Qiang Wang, Hua-Jie Yang, Xi-Mao Wu, Chun-He Li, Zhe-Feng Zhang, Xiao-Wu Li

**Affiliations:** 1 Department of Materials Physics and Chemistry, School of Materials Science and Engineering, Northeastern University, Shenyang 110819, China; jphou13s@imr.ac.cn; 2 Shenyang National Laboratory for Materials Science, Institute of Metal Research, Chinese Academy of Sciences, Shenyang 110016, China; gmwang@imr.ac.cn (Q.W.); hjyang@imr.ac.cn (H.-J.Y.); zhfzhang@imr.ac.cn (Z.-F.Z.); 3 Electric Power Research Institute of Liaoning Electric Power Co., Ltd., Shenyang 110006, China; xmwu69@yahoo.com (X.-M.W.); chli64@yahoo.com (C.-H.L.)

**Keywords:** commercially pure aluminum conductor, cold drawing, tension–tension fatigue, grain boundary migration, fracture

## Abstract

Fatigue properties and cracking behavior of cold-drawn commercially pure aluminum wires (CPAWs) widely used as the overhead transmission conductors were investigated. It was found that the fracture surface of the CPAWs shows an obvious four-stage fracture characteristic, i.e., crack initiation, planar crack propagation, 45°-inclined crack propagation and final rapid fracture. The crack growth mechanisms for the CPAWs were found quite different from those for the conventional coarse-grained materials. The cracks in the CPAWs firstly grow along the grain boundaries (Stage I crack growth), and then grow along the plane of maximum shear stress during the last stage of cycling (Stage II crack growth), leading to the distinctive fracture surfaces, i.e., the granular surface in the planar crack propagation region and the coarse fatigue striations in the 45°-inclined crack propagation region. The grain boundary migration was observed in the fatigued CPAWs. The increase in fatigue load enhances the dislocation recovery, increases the grain boundary migration rate, and thus promotes the occurrence of softening and damage localization up to the final failure.

## 1. Introduction

With increasing demand of electricity in the past years, there has been a rapid development in the power industry. As one of the key metallic materials applied in the overhead transmission lines, commercially pure (CP) aluminum is more suitable than other metals considering its economical factors, mechanical properties and electrical performances [1,2]. For instance, the external-layers of the aluminum conductor steel reinforced (ACSR) and the aluminum conductor alloy reinforced (ACAR) widely applied to overhead power lines are commonly made of CP aluminum. A number of investigations have been mainly focused on the strength, electrical conductivity and deformation behavior of the CP aluminum and aluminum alloys [2,3,4,5,6,7,8,9]. However, the fatigue performance of these materials is also a critical problem, which should be further studied. In practical conditions, the commercially pure aluminum wires (CPAWs) may suffer a tensile load from wind, ice and its deadweight, and the CPAWs may thus become quite dangerous under cyclic loads due to the wind-induced swing of the overhead transmission lines [10]. As a result, the fatigue lives of the CPAWs under different stress amplitudes should be estimated for determining their security service conditions.

Revealing the fatigue fracture mechanisms of the CP aluminum by analyzing fracture surfaces, which are closely related to the loading conditions and the material properties, is helpful for improving engineering failure analysis and understanding fracture behavior. In general, the fatigue fracture surfaces of most metallic materials consist of three different regions, i.e., crack initiation region, crack propagation region and final rapid fracture region [11,12,13,14]. The fatigue properties and microstructures of aluminum alloys have attracted many attentions [15,16,17,18,19,20], and many well-developed mechanisms for the fatigue crack growth have been summarized [21]; for example, Forsyth [22] plotted two stages for fatigue crack growth. McDowell et al. [23] and Suresh [24] described a four-stage crack growth mechanism based on the microstructural observations, including incubation, microstructurally small crack growth, physically small crack growth and long crack growth. However, the fatigue and fracture behaviors of the CPAWs are rarely reported. Azevedo et al. [10] found that there are three types of fracture surfaces for the pure aluminum conductors subjected to different bending amplitudes, i.e., planar-type surface, V-type surface and 45°-type surface.

In the present work, the stress-life (S-N) curve of the CPAW was obtained by tension–tension fatigue tests under different maximum stress amplitudes. The relation between the fracture features and the stress amplitude was established by scanning electron microscope (SEM) examinations. Furthermore, the microstructure evolution in the fatigued CPAWs was examined using transmission electron microscopy (TEM) to reveal the fracture mechanism of CPAWs.

## 2. Experimental Section

**Figure 1:**
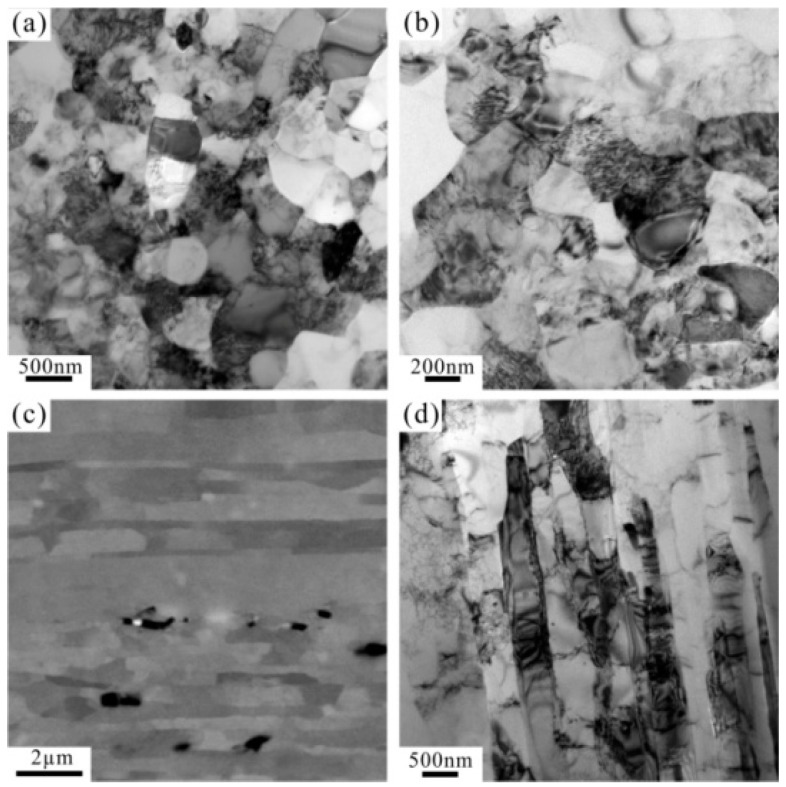
TEM and SEM images of the microstructures on (**a**,**b**) the cross-section and (**c**,**d**) the longitudinal-section of the unfatigued commercially pure aluminum wires (CPAWs).

The chemical composition of the CP aluminum (wt %) is aluminum ≥ 99.7 and impurity ≤ 0.3. A CPAW with a diameter of 3.25 mm was manufactured using a bull block drawing machine by cold drawing for 9 passes from the original aluminum rod. The microstructure of the unfatigued CPAW is shown in Figure 1. There are many dislocations in most grains induced from the cold-drawing process. The grains are equiaxed on the cross-section (Figure 1a,b) but elongated along the drawing direction on the longitudinal-section (Figure 1c,d). The uniaxial tensile tests of the cold-drawn CPAWs with a gauge length of 150 mm and a diameter of 3.25 mm were carried out on a Shimadzu AG-X testing machine. The tensile specimens were tested at room temperature with a constant strain rate of 1.0 × 10^−3^ s^−1^ and the tensile axis was parallel to the drawing direction.

**Figure 2:**
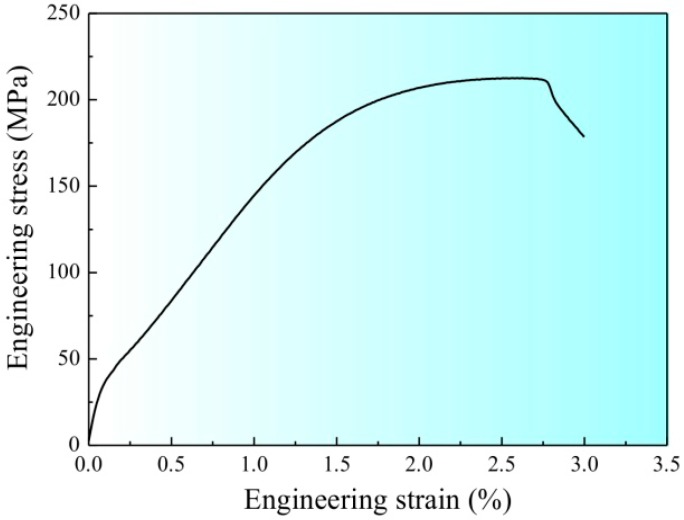
The tensile stress–strain curve of the CPAWs.

**Figure 3:**
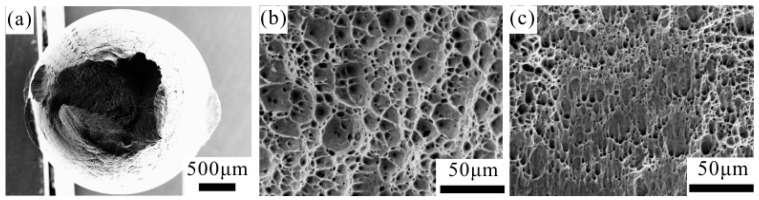
SEM images of the tensile fracture surface of the unfatigued CPAWs: (**a**) overall view; and (**b**,**c**) high-magnification images showing the dimple features.

Figure 2 shows the tensile stress–strain curve of the CPAWs, which exhibits typical mechanical characteristic of the cold-worked materials. The yield strength and the ultimate tensile strength of the CPAWs are ~203.5 MPa and ~212.5 MPa, respectively. The strengthening mechanisms of the CPAWs have been explained by the formation of high-density dislocations, <111> textures and high-angle grain boundaries in our previous study [6]. The fracture elongation of the CPAWs is only about 2.8%. The corresponding fracture surface features of the CPAWs are shown in Figure 3. Shear lip, equiaxed dimples and shear dimples were observed on the surface of the tensile specimen, showing a ductile fracture feature.

The cylindrical fatigue specimens with a gauge length of 13 mm and a diameter of 3.25 mm machined from the CAPWs were fatigued on an Instron 8871 testing machine under tension–tension loading at room temperature with a stress ratio (*R*) of 0.1 and a frequency of 40 Hz. The maximum stress amplitudes used in this study are 90 MPa, 100 MPa, 140 MPa, 160 MPa and 200 MPa, respectively. After fatigue tests, the fracture morphology of the specimen was observed using ZEISS SUPRA 35 SEM (ZEISS, Oberkochen, Germany). TEM samples were cut from the cross section of the CPAWs, ground to a thickness of ~0.05 mm and then twin-jet electropolished using a solution of 20% perchloric acid and 80% methanol in volume. TEM foils were examined using an FEI Tecnai F20 microscope (FEI, Hillsboro, OR, USA) operating at 200 kV. The linear intercept method was adopted to measure the average grain sizes of the CPAWS, and more than 200 grains were measured for each sample fatigued at different stress amplitudes.

## 3. Results and Discussion

### 3.1. S-N Curve

**Figure 4:**
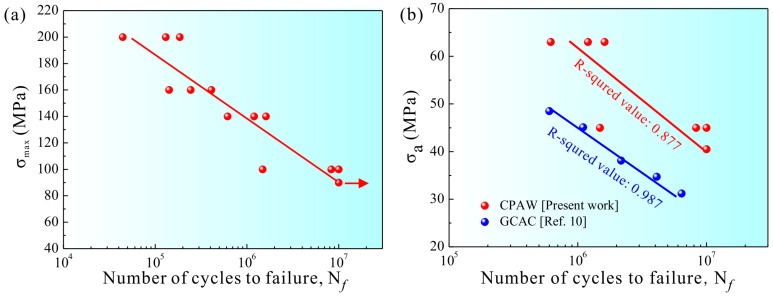
(**a**) S-N curve plotted by the maximum stress amplitude against the number of cycles to failure for the CPAWs fatigued at tension–tension loading with a stress ratio of 0.1; and (**b**) comparison of the S-N curves of the CPAW in the present work and the Grosbeak cable arrangement conductor (GCAC) in Ref. [10].

The maximum stress amplitude–fatigue life (S-N) curve of the CPAW is shown in Figure 4a. The fatigue life increases as the maximum stress amplitude decreases. The S-N curve of the CPAW approximately exhibits a linear relationship in double-logarithmic coordinates. Here, if the specimen survived 10^7^ cycles at a certain maximum stress amplitude level, the corresponding stress amplitude was defined as the fatigue strength, which was estimated to be around 90 MPa for the CPAW.

Figure 4b shows the comparison of the S-N curves for the CPAW and the Grosbeak cable arrangement conductor (GCAC) [10]. It should be mentioned that the surface roughness and the residual stress would influence the fatigue strength. However, the industrial manufacturing route of the CPAW is similar to that of the GCAC [10]. Here, such influencing factors can thus be neglected for the comparison of fatigue properties for these two kinds of materials. Clearly, the S-N curve for the CPAW lies significantly higher in terms of fatigue life than that for the GCAC. It essentially means that the CPAW presents much higher fatigue strength than the GCAC.

### 3.2. Fracture Characteristics

Fracture surfaces of the specimens fatigued at different maximum stress amplitudes were observed to reveal the fatigue fracture behavior of the CPAW.

**Figure 5:**
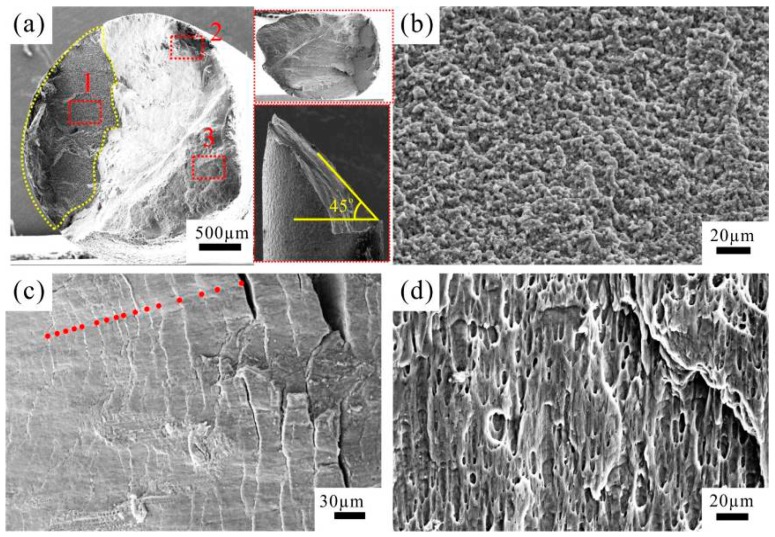
SEM images of fractographic surface of the specimen fatigued at 100 MPa: (**a**) overview; and (**b**–**d**) high-magnification images corresponding, respectively, to regions 1, 2 and 3 in (**a**).

Figure 5 shows the fracture surface features of the CPAW fatigued at 100 MPa. It is clear that the fracture surface exhibits two distinctive regions, i.e., a planar region vertical to the loading axis, and a 45°-inclined region with the loading axis (Figure 5a). The crack initiation site was found at the surface of the specimen. As shown in Figure 5b (a high-magnification image of the region 1 in Figure 5a), the granular surface in the planar region is smooth and flat, which is similar to that of the fatigued ultrafine-grained copper [25]. The planar region is generally considered as the crack propagation region. However, the fatigue striation, a typical feature in the crack propagation region, was not observed in this region. It is interesting to find that the striation-like morphology (Figure 5c) was formed in the 45°-inclined region (region 2 in Figure 5a). The spaces of the striation-like morphology are in the range of 9.1 to 25.6 μm, indicating that the fatigue crack growth rate *da*/*dN* is very high (9.10 × 10^−6^ m/cycle −2.56 × 1^−5^ m/cycle), because the positions of the striation-like morphology are very close to the final rapid fracture region. Figure 5d shows a dimple feature in the final rapid fracture region corresponding to region 3 in Figure 5a, demonstrating that the final fracture mode of the CPAW is ductile fracture.

**Figure 6:**
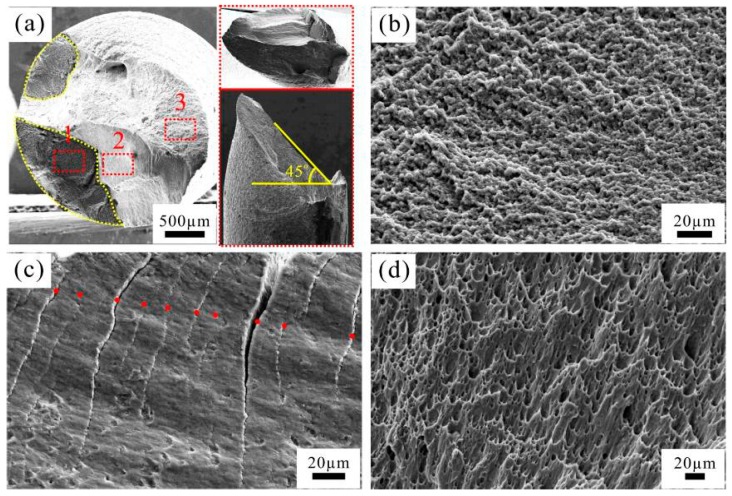
SEM images of fractographic surface of the specimen fatigued at 140 MPa: (**a**) overview; and (**b**–**d**) high-magnification images corresponding, respectively, to regions 1, 2 and 3 in (**a**).

The fracture surface of the CPAW fatigued at 140 MPa is shown in Figure 6. The fracture surface also consists of the planar region and the 45°-inclined region. However, the size of the planar region is smaller than that of the specimen fatigued at 100 MPa. In addition, two separate planar regions can be observed in Figure 6, indicating that there are at least two crack initiation sites on the surface of the specimen. Similarly, the granular surface in the planar region (Figure 6b), striation-like morphology in the whole 45°-inclined region (Figure 6c), and dimples in the final rapid fracture region (Figure 6d) were also observed.

**Figure 7:**
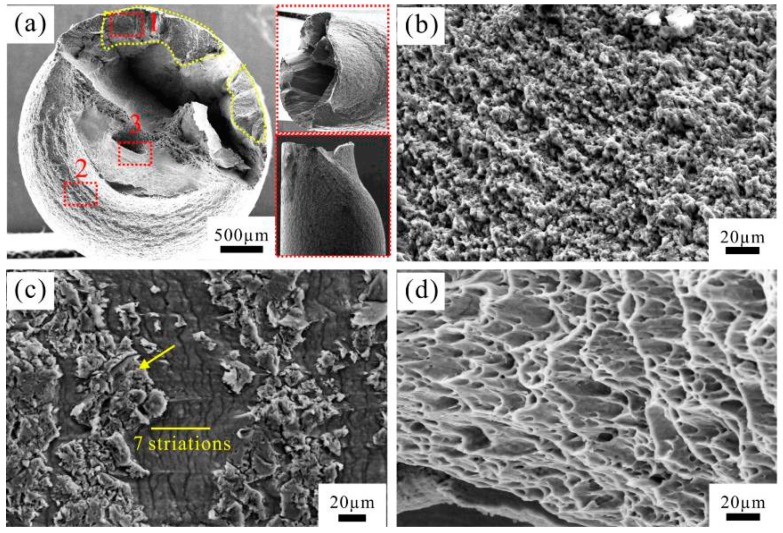
SEM images of fractographic surface of the specimen fatigued at 160 MPa: (**a**) overview; and (**b**–**d**) high-magnification images corresponding, respectively, to regions 1, 2 and 3 in (**a**).

On cycling at an increased stress amplitude of 160 MPa, the fracture surface exhibits a morphology similar to the V-type fractography [10], as shown in Figure 7a. There are two crack propagation regions (regions 1 and 2 in Figure 7a) with different features. No striations were observed in the planar region (Figure 7b); however, typical fine fatigue striations were found in another crack propagation region (Figure 7c). The average striation spacing is about 6.70 μm less than those of the specimens fatigued at 100 MPa and 140 MPa. Dimples were found in the final rapid fracture region as expected (Figure 7d).

**Figure 8:**
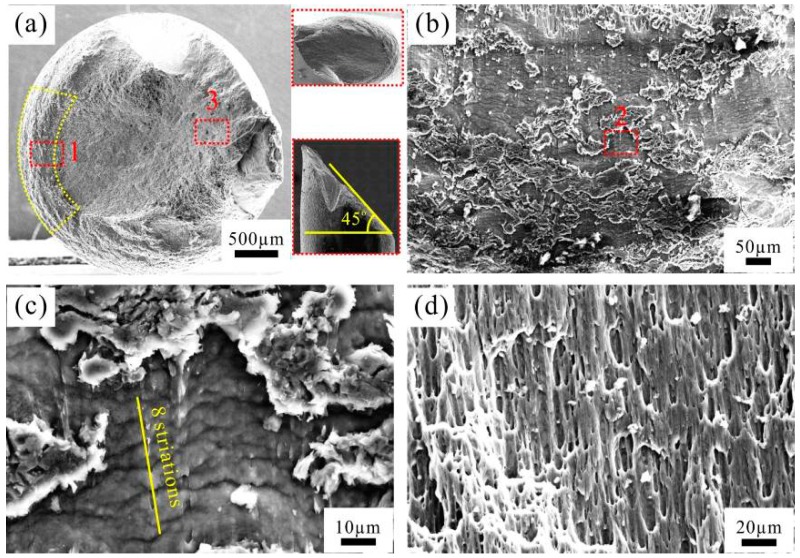
SEM images of fractographic surface of the specimen fatigued at 200 MPa: (**a**) overview; (**b**) high-magnification image of region 1 in (**a**); (**c**) further-magnified image of region 2 in (**b**); and (**d**) high-magnification image of region 3 in (**a**).

As the stress amplitude increases to the highest value of 200 MPa adopted, which is very close to the ultimate tensile strength of the CPAW (~212.5 MPa), nearly no planar region was detected on the fracture surface (Figure 8a). In this case, the fracture surface shows a characteristic of pure shear fracture, which is quite similar to that under uniaxial tensile tests. Figure 8c shows that the average spacing of the fine fatigue striations in the crack propagation region (Figure 8b) is about 6.40 μm, which is almost equal to that of the specimen fatigued at 160 MPa. The final rapid fracture region also shows a dimple-type ductile fracture feature (Figure 8d).

**Figure 9:**
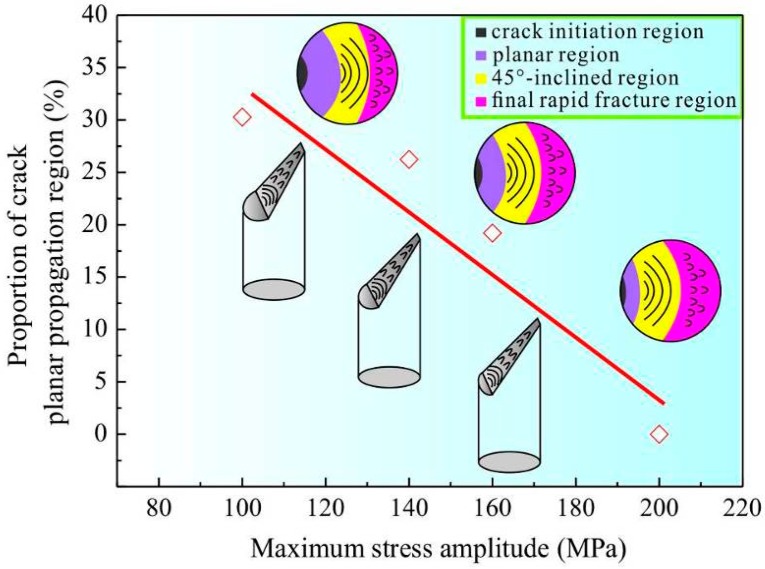
Illustration of the four-stage region characteristic (crack initiation region, planar crack propagation region, 45°-inclined crack propagation region and final rapid fracture region) of the fracture surface morphology in the fatigued CPAWs. Note that different colors represent different regions. The proportion of the planar crack propagation region decreases with increasing fatigue stress. In addition, coarse fatigue striations and dimples were found in the 45°-inclined crack propagation region and the final rapid fracture region, respectively.

Figure 9 illustrates the evolution of fracture surfaces of the CPAWs fatigued with maximum stress amplitude. The fracture surfaces of the specimens fatigued at relatively low stress amplitudes (100 MPa, 140 MPa and 160 MPa) exhibit typical four-stage region, i.e., crack initiation region, planar region, 45°-inclined region and final rapid fracture region. The planar region and 45°-inclined region are considered as the crack propagation region. Therefore, the crack propagation region of the fatigued CPAW exhibits a two-stage feature, a planar crack propagation region and a 45°-inclined crack propagation region. Here, the proportion of the planar crack propagation region is defined as the ratio of the planar region area to the final cross-section area of the specimen. It should be noted that the effect of necking on the cross-section area is taken into consideration. Obviously, the proportion of the planar crack propagation region decreases with increasing stress amplitude (Figure 9). The striation-like morphology with the spacing of 9.05–25.56 μm was found in the 45°-inclined crack propagation region, indicating a quite high fatigue crack growth rate. Relatively fine fatigue striations with a spacing of about 6.50 μm were found in the crack propagation region of the specimens fatigued at 160 MPa and 200 MPa. The formation of striations was convinced to be associated with crack tip retardation and blunting [26]. Dimples were found in the final rapid fracture region of all the specimens, indicating a ductile fracture mode.

**Figure 10:**
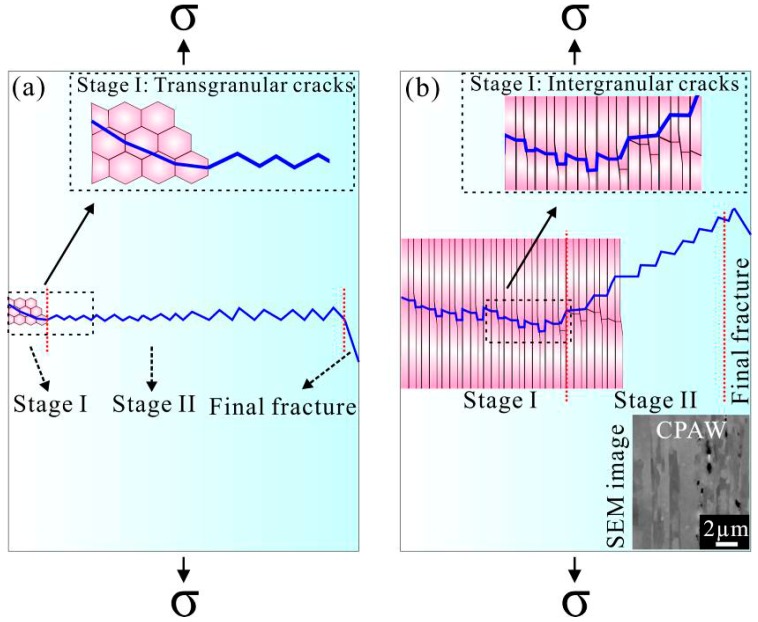
Illustration of the crack growth stages in: (**a**) conventional coarse-grained materials; and (**b**) fine-grained CPAW in the present work under cyclic loading. For the coarse-grained materials, the cracks grow along the planes of maximum shear stress and on the planes normal to the direction of maximum principle stress in stage I and stage II, respectively. For the CPAW, the cracks grow along the grain boundaries and along the planes of maximum shear stress in stage I and stage II, respectively.

The different fracture surfaces are the demonstrations of the different mechanisms in various stages of their evolution. Figure 10 illustrates the different stages of crack growth in the conventional coarse-grained materials and the present CPAW under cyclic loading. As shown in Figure 10a, for the conventional coarse-grained materials, the crack growth can be divided into two stages, namely, the cracks grow along the planes of maximum shear stress in stage I, showing a transgranular slip cracking characteristic, and then the cracks propagate on the planes normal to the direction of maximum principle stress until the final failure occurs [22]. However, the crack growth mechanism for the CPAW is quite different from that for the coarse-grained materials. The CPAW consists of elongated fine grains; therefore, the cracks firstly grow along the grain boundaries (GBs) where an incompatibility of plastic deformation in adjacent grains is concentrated [25], showing an intergranular cracking characteristic and a relevant granular fracture surface in the planar region. As cycling continues, the effective loading area of the CPAW decreases, and then the cracks prefer to grow along the plane of maximum shear stress as the tensile stress exceeds the load-carrying capacity of the remaining area of the CPAW, resulting in the formation of the 45°-inclined crack propagation region. Certainly, as the adopted stress amplitude, e.g., 200 MPa, is close to the ultimate tensile strength of the CPAW, the planar region almost disappears, as seen in Figure 8. The 45°-inclined crack propagation region usually occurs during the last stage of cycling, leading to a large striation spacing and a high fatigue crack growth rate. As a result, the fatigue life of the CPAW is mainly determined by the number of cycles accumulated in the intergranular cracking growth stage. That is to say, the fatigue life increases with the area of planar region, as shown in Figure 9. It can be concluded that the crack growth mechanisms for the CPAW are remarkably different from those for the conventional coarse-grained materials, and thus distinctive fracture surface morphologies are presented in the CPAW (Figure 5, Figure 6, Figure 7 and Figure 8).

### 3.3. Deformation Microstructures

GB is considered as an important microstructure to impede the dislocation motion and result in an increase in tensile strength [27,28,29]. The migration of GBs can occur as suffering from static or dynamic loading, thus influencing the mechanical properties of a material [30]. Besides, the GB migration behavior in different materials under various temperatures and loading conditions has been widely reported [31,32,33,34]. The drive force of GB migration can be attributed to the different free energies across the GB due to several factors such as cold-working, boundary curvature, misorientation, or non-uniform stress states [30].

**Figure 11:**
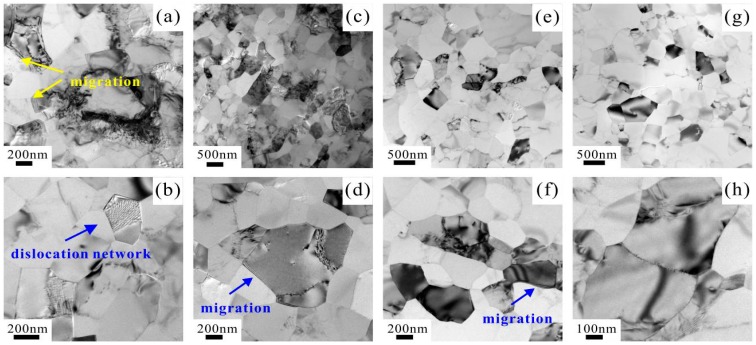
TEM images of microstructures of the specimens fatigued at: (**a**,**b**) 90 MPa; (**c**,**d**) 100 MPa; (**e**,**f**) 140 MPa; and (**g**,**h**) 200 MPa.

Figure 11a–d shows the microstructures of the specimens fatigued at relatively low stress amplitudes of 90 MPa and 100 MPa. It can be seen that dislocations still exist in a small part of grains. However, most grain interiors become clean due to the recovery of dislocations under the action of cyclic loading. Specifically, the dislocation networks were found in several grains, as shown in Figure 11b, providing a convincing evidence for the occurrence of dislocation recovery. Furthermore, as shown in Figure 11a, the GB migration was mostly found between the two grains with different contrasts under bright field image, indicating that these grains are highly misoriented. That is to say, the GB migration occurs more easily when the misorientation of neighboring grains is high, which is quite consistent with the results reported by Badirujjaman et al. [33]. The microstructures of the specimens fatigued at 140 MPa and 200 MPa are shown in Figure 11e–h. Obviously, there are almost no dislocations in all grains, indicating that the recovery of dislocations has been fully developed. The GB migration can be seen in a few of GBs, as shown in Figure 11e,f. By contrast, the GB migration nearly disappears as the stress amplitude increases to 200 MPa (Figure 11g,h), showing a typical microstructure involving grain growth.

The GB migration plays an important role in grain growth, and governs the microstructure and the texture evolution in crystalline materials [30]. In general, one of the direct consequences of the GB migration is the growth of grains [30,35]. As shown in Table 1, the average grain size for the specimen fatigued at 90 MPa is equal to that for the unfatigued specimen, followed by a slight increase in grain size with increasing fatigue load. The GB migration requires a driving force induced by the gradient of free energy on either side of the GB, which can be obtained from the stored energy of cold-working, boundary curvature, anisotropy of physical properties, etc. Here, the stored energy induced by cold-drawing can be released in the form of dislocation recovery during cyclic loading. As evidenced by Figure 11, with increasing fatigue load, the dislocation recovery becomes enhanced and the GB migration rate increases, promoting the occurrence of softening and damage localization up to the final failure.

## 4. Conclusions

The fatigue properties, fatigue fracture behavior and microstructure evolution of the CPAWs used for overhead power lines were investigated under cyclic tension–tension loading. The following conclusions can be drawn:

1. The fatigue strength defined at 10^7^ cycles for the CPAW is determined to be ~90 MPa under tension–tension fatigue tests with a stress ratio *R* = 0.1.
2. Fracture surfaces of the CPAWs fatigued at different stress amplitudes exhibit a four-stage characteristic, i.e., crack initiation region, planar crack propagation region, 45°-inclined crack propagation region and final rapid fracture region. The proportion of the planar crack propagation region decreases with increasing fatigue load. The crack growth mechanism for the CPAW is quite different from that for the coarse-grained materials. The cracks in the CPAWs firstly grow along the grain boundaries, and then propagate along the plane of maximum shear stress during the last stage of cycling, showing the distinctive crack growth mechanisms and fracture surfaces, i.e., the granular surface in the planar crack propagation region (Stage I) and the coarse fatigue striations in the 45°-inclined crack propagation region (Stage II).
3. The GB migration was observed in the fatigued CPAWs. With increasing fatigue load, the dislocation recovery becomes more evident, and the GB migration rate increases, promoting the occurrence of softening and damage localization and resulting in the final failure.

## Figures and Tables

**Table 1:** The average grain size of the commercially pure aluminum wires (CPAWs) fatigued at different stress amplitudes.

**Maximum Stress Amplitude (MPa)**	0	90	140	200
**Average Grain Size (μm)**	0.39	0.39	0.41	0.45
